# Characterization of the complete chloroplast genome of *Asparagus setaceus*

**DOI:** 10.1080/23802359.2019.1643798

**Published:** 2019-07-18

**Authors:** Jia-Rong Li, Shu-Fen Li, Jin Wang, Ran Dong, Hong-Wei Zhu, Ning Li, Chuan-Liang Deng, Wu-Jun Gao

**Affiliations:** College of Life Sciences, Henan Normal University, Xinxiang, China

**Keywords:** *Asparagus setaceus*, chloroplast genome, high-throughput sequencing, phylogenetic analysis

## Abstract

*Asparagus setaceus* is an important ornamental plant and is important to study the sex chromosome evolution in genus *Asparagus*. Here the complete chloroplast (cp) genome sequence of *A. setaceus* was determined. The cpDNA is 156,978 bp in length, contains a large single copy region of 85,311 bp and a small single copy region of 18,641 bp, which was separated by a pair of inverted repeat regions of 26,513 bp each. The cp genome contains 132 genes, including 88 protein-coding genes, 6 ribosomal RNA genes, and 38 transfer RNA genes. Phylogenetic analysis indicates that *A. setaceus* is closely related to *Asparagus officinalis* and *Asparagus schoberioides*.

*Asparagus setaceus* is an important ornamental plant and belongs to genus *Asparagus*. The genus *Asparagus* consists of both dioecious and hermaphrodite plants, which made *Asparagus* an ideal genus for studying the sex chromosome origin and evolution. The transfer of chloroplast DNA into nuclear genomes form nuclear integrants of plastid DNAs (NUPTs), which likely play important roles in the sex chromosome evolution (VanBuren and Ming 2013; Steflova et al. 2014). As a hermaphrodite plant, *A. setaceus* is an important species for comparative studies of the sex chromosome evolution of the genus *Asparagus*. In this study, we assembled and characterized the complete chloroplast DNA of *A. setaceus*. The data presented here can provide a useful resource for further study of sex chromosome evolution of genus *Asparagus* and facilitate analysis of the evolution and genetics of this species and other species in the same genus.

Total genomic DNA was isolated from fresh leaves collected from an individual of *A. setaceus* grown in the greenhouse of Henan Normal Univeristy (Xinxiang, China; 113°91′E, 35°31'N). The voucher specimen (Li S.F., No. 2017110501) was deposited at the Biological Specimen Depository of Henan Normal University. Total DNA was used for the shotgun library construction and the subsequent high-throughput sequencing on the GridION X5 sequencing platform. A total of 123.6 Gb raw reads were obtained, quality-filtered, and assembled using Minimap2 (Li 2018) with the reference sequence of *Asparagus officinalis* (GenBank: LN896355.1). The chloroplast (cp) genome was annotated using DOGMA (Wyman et al. 2004) and corrected manually. The annotated cp genome sequence has been deposited into the GenBank with the accession number MK950153.

The complete cp genome of *A. setaceus* was 156,978 bp in length, consisting of a pair of inverted repeat regions of 26,513 bp each, a large single copy region of 85,311 bp, and a small single copy region of 18,641 bp. The overall GC content of the cp genome was 37.5%. A total of 132 genes were annotated, including 38 tRNA, 6 rRNA, and 88 protein-coding genes.

To confirm the phylogenetic position of *A. setaceus*, the complete chloroplast sequences of 13 species of Liliaceae (Monocotyledoneae) and 5 species of other Monocotyledons were aligned using MAFFT v.7 (Katoh and Standley 2013) and maximum-likelihood (ML) analysis was conducted using FastTree 2 (Price et al. 2010). The ML tree showed that *A. setaceus* is closely realted to *A. officinalis* and *Asparagus schoberioides* (Figure 1).

**Figure 1.**
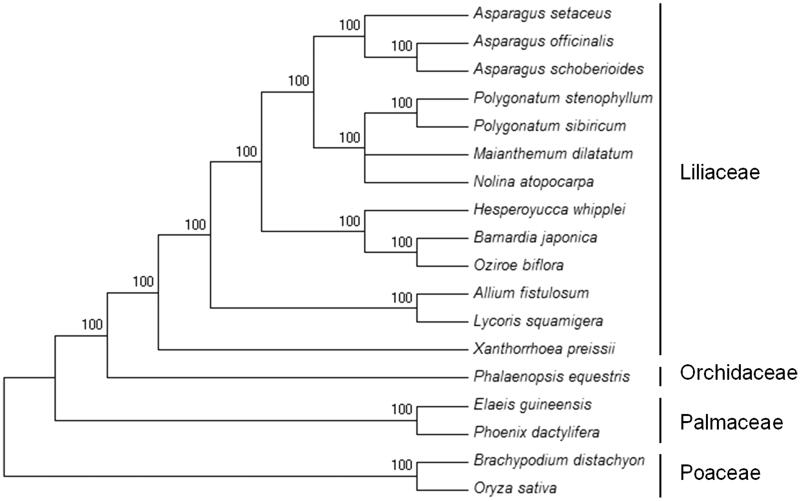
Maximum-likelihood (ML) phylogenetic tree based on 18 complete chloroplast genomes. Accession numbers: *Asparagus setaceus* (MK950153); *Asparagus officinalis* (LN896355.1); *Asparagus schoberioides* (NC_035969.1); *Polygonatum stenophyllum* (KX822773.1); *Polygonatum sibiricum* (NC_029485.1); *Maianthemum dilatatum* (NC_039133.1); *Nolina atopocarpa* (KX931462.1); *Hesperoyucca whipplei* (KX931459.1); *Barnardia japonica* (MH287351.1); *Oziroe biflora* (KX931463.1); *Allium fistulosum* (NC_040222.1); *Lycoris squamigera* (NC_040164.1); *Xanthorrhoea preissii* (KX822774.1); *Phalaenopsis equestris* (NC_017609.1); *Elaeis guineensis* (NC_017602.1); *Phoenix dactylifera* (NC_013991.2); *Brachypodium distachyon* (NC_011032.1); *Oryza sativa* (NC_031333.1).
